# The Role of the Lateral Habenula in Punishment

**DOI:** 10.1371/journal.pone.0111699

**Published:** 2014-11-03

**Authors:** Philip Jean-Richard Dit Bressel, Gavan P. McNally

**Affiliations:** School of Psychology, University of New South Wales, Sydney, NSW, Australia; Medical University of South Carolina, United States of America

## Abstract

The lateral habenula (LHb) is a small epithalamic structure that projects via the fasciculus retroflexus to the midbrain. The LHb is known to modulate midbrain dopamine (DA) neurons, including inhibition of ventral tegmental area (VTA) neurons via glutamatergic excitation of the GABAergic rostromedial tegmental nucleus (RMTg). A variety of lines of evidence show activity in LHb and the LHb-RMTg pathway is correlated with, and is sufficient to support, punishment learning. However, it is not immediately clear whether LHb is necessary for punishment. Here we used a within-subjects punishment task to assess the role of LHb in the acquisition and expression of punishment as well as in aversive choice. Rats that pressed two individually presented levers for pellet rewards rapidly suppressed responding to one lever if it also caused footshock deliveries (punished lever) but continued pressing a second lever that did not cause footshock (unpunished lever). Infusions of an AMPA receptor antagonist (NBQX) into LHb had no effect on the acquisition or expression of this punishment, or on aversive choice, but did increase locomotion. Infusion of the sodium channel blocker bupivacaine likewise had no effect on expression of punishment. However, infusion of the calcium channel blocker mibefradil did affect expression of punishment by significantly decreasing the latency with which rats responded on the punished lever and significantly increasing unpunished lever-pressing. Taken together, these findings indicate that the LHb plays a limited role in punishment, influencing only latency to respond. This role is linked to calcium channel permeability and not AMPA receptor or sodium channel permeability.

## Introduction

The lateral habenula (LHb) is a small epithalamic structure that projects via the fasciculus retroflexus to the midbrain [1]. The LHb is known to modulate midbrain dopamine (DA) neurons, including inhibition of ventral tegmental area (VTA) neurons via glutamatergic excitation of the GABAergic rostromedial tegmental nucleus (RMTg) [2] and possible excitation of these neurons via a direct projection [3]. The LHb has been implicated in a variety of functions including motor suppression, cognition, pain, stress, and reward, as well as regulation of reproductive behaviour, circadian rhythms and metabolism [4]–[6].

Of particular interest to the experiments reported here is the claim that LHb is important for aversive motivational value and punishment [6]–[8]. Two primary lines of evidence have typically been invoked to support this possibility. First, recording studies in rhesus monkeys have shown that LHb and RMTg neurons are phasically excited by unexpected aversive events and reward omissions, as well as by cues that predict those outcomes. These phasic excitations are closely followed by a phasic inhibition of midbrain DA neuronal firing [2]. These results have been interpreted as LHb neuronal coding of aversive outcome values and consequently suppressing motor behavior and reward seeking. Second, focal electrical or optogenetic stimulation of the LHb-RMTg-VTA pathway is aversive and can act as a punisher [8]–[11]. For example, Stamatakis and Stuber [8] reported that ChR2 stimulation of LHb terminals in the RMTg supported both active and passive place avoidance learning in mice, and negatively reinforced as well as positively punished nosepoking behaviour in mice.

These findings show that activity in LHb and the LHb-RMTg pathway is correlated with, and is sufficient to support, punishment learning. However, it is not immediately clear whether LHb is necessary for punishment. Although recent studies support this possibility broadly for LHb encoding of aversive motivational value, showing for example that lesions of the rat LHb/fasciculus retroflexus attenuate the aversive motivational properties of cocaine [12] as well as ethanol [13] and that reversible inactivations of LHb impair avoidance learning [14], the requirement of LHb for the acquisition and expression of punishment behaviour remains unknown.

The aim of this experiment was to study the role of the LHb in the acquisition and expression of punishment in rats. Rats received bilateral cannulation of the LHb permitting reversible inactivation using the AMPA/kainate receptor antagonist NBQX. NBQX was used due to previous findings showing that aversion-related signals are conveyed to the LHb from the basal ganglia and that transmission via this pathway could be blocked by NBQX [15], [16]. Rats were then subjected to a multi-stage procedure assessing punishment as well as aversive choice (**Table 1**). The initial phase assessed the role of LHb in the acquisition of punishment. The next phases investigated the role of LHb in expression of punishment and/or instrumental aversive choice. In order to assess a broader range of LHb manipulations, the effect of LHb infusions using sodium and calcium channel blockers (bupivacaine and mibefradil, respectively) on expression of punishment were also examined. Finally, rats were assessed for the effects of LHb inactivation on locomotor activity.

**Table 1:** Experimental design.

	Phase I	Phase II	Phase III	Phase IV
	*Punishment*	*Punishment*		
*Pretraining*	*Acquisition*	*Expression*	*Channel Blockers*	*Locomotion*
(*n* = 16)	(*n* = 16)	(*n* = 15)	(*n* = 10)	(*n* = 16)
	**Sal**:		**Bup/Mib:**	
	L1– food; shock		L1– food; shock	
	L2– food	**Sal/NBQX:**	L2– food	
L1– food		L1– food; shock	***Aversive Choice***	**Sal/NBQX**
L2– food	**NBQX**:	L2– food	(*n* = 6)	
	L1– food; shock		**Sal/NBQX:**	
	L2– food		L1 & L2– food	

Food was a single 45 mg pellet on a VI30s (lever-press training, Phase I, Phase II, Phase III channel blockers) or VI60s (aversive choice); shock was a 0.5 s, 0.5 mA footshock on an FR-10 schedule.

## Materials and Methods

### Subjects

Subjects were 17 experimentally naive male Sprague Dawley rats (300–380 g) obtained from a commercial supplier (Animal Resources Centre, Perth, Australia). Rats were housed in groups of four in plastic cages and maintained on a 12 hr light-dark cycle (lights on at 7∶00 A.M.). The procedures used were approved by the Animal Care and Ethics Committee at the University of New South Wales and were conducted in accordance with the National Institutes of Health (NIH) *Guide for the Care and Use of Laboratory Animals* (NIH Publications No. 80-23, revised 1996).

### Apparatus

All behavioural training was conducted in a set of eight identical experimental chambers (24 cm [length] × 30 cm [width] × 21 cm [height]; Med Associates Inc., Georgia, VT, USA). Each chamber was enclosed in sound- and light-attenuating cabinets (55.9 cm [length] × 35.6 cm [width] × 38.1 cm [height]) and fitted with fans for ventilation and background noise. The chambers were made up of a Perspex rear-wall, ceiling and hinged front-wall, and stainless steel sidewalls. The chamber floors were made of stainless steel rods (4 mm in diameter) spaced 15 mm apart. Each chamber stood 35 mm above a tray of corncob bedding. A recessed magazine (3 cm in diameter) within a 4 cm × 4 cm hollow in the right-side chamber wall received pellets from an external automatic hopper. Infrared photocells detected entries into the magazine.

Two retractable levers were placed either side of the magazine. In both experiments, a 45 g grain pellet, which was delivered to the magazine from the external hopper, served as the reward. The punisher was a 0.5 sec, 0.5 mA footshock delivered through the grid floor. All chambers were connected to a computer with Med-PC IV software (Med Associates, St Albans, VT, USA), which controlled lever, pellet and shock presentations and recorded the lever-presses and magazine entries.

Locomotor activity was assessed in Plexiglas chambers (Med Associates, St Albans, VT, USA) 43.2 cm (width) × 43.2 cm (length) × 30.5 cm (height) for 40 min. Movement was tracked through the use of three 16 beam infrared arrays. Infrared beams were located on both the X and Y-axes for positional tracking.

### Procedure

#### Surgery

Rats were anaesthetized with 1.3 ml/kg ketamine (100 mg/ml; Ketapex; Apex Laboratories, Sydney, Australia) and 0.2 ml/kg muscle relaxant, xylazine (20 mg/ml; Rompun; Bayer, Sydney, Australia) (i.p.) and placed in stereotaxic apparatus (Model 900, Kopf, Tujunga, CA), with the incisor bar maintained at approximately 3.3 mm below horizontal to achieve a flat skull position. 26 gauge guide cannulae (6 mm in length; Plastics One, Virginia) were implanted bilaterally into the LHb according to the coordinates AP: −3.8, ML: ±0.8, DV: −4.8 mm from bregma. The guide cannulae were fixed in position with dental cement and jeweller’s screws. Dummy cannulae were kept in the guide at all times except during microinjections. Rats were allowed to recover for 5 days prior to the start of the experimental procedure.

#### Food deprivation

Commencing five days after surgery and persisting for the duration of the experiment, rats received daily access of 10–15 g of food (to attain and then maintain a weight of approximately 90% compared to immediately prior food deprivation) and unrestricted access to water in their home cages. It was established in pilot experiments that this deprivation schedule was sufficient to motivate continuous unpunished lever-pressing but only a moderate amount of mildly-punished lever-pressing.

#### Lever-press training

Three days after commencement of food deprivation, rats were placed in the experimental chambers for 30 mins to acclimatise and were then given lever-press training, which consisted of two levers (left and right) being extended and reinforced with grain pellets on a fixed ratio-1 (FR-1) schedule for 1 h, or until each lever had been pressed 25 times each (each lever would retract after 25 presses). Houselights were on throughout the session. All rats received another day of lever-press training, and any rats that did not acquire lever-pressing were manually shaped until lever-pressing was acquired. All rats were then given 7 days of lever-press training. Levers were presented individually in an alternating pattern so that one lever was extended for 5 mins while the other lever was retracted. After 5 mins the extended lever was retracted and the retracted lever was extended, such that each lever was always presented on its own. This alternation occurred throughout the 40 min session. Both levers were reinforced with a pellet on a VI-30 s schedule (a lever-press causes immediate pellet delivery on average every 30 sec).

#### Punishment Acquisition

On Days 1–5, rats were trained and tested in the punishment task. Punishment sessions were identical to acquisition sessions, except that a designated lever was also punished with a 0.5 s, 0.5 mA footshock on an FR-10 schedule. The same lever (left or right) was designated as “punished” throughout the experiment for each rat but which lever was designated as punished was counterbalanced between rats. Immediately before the first 2 days of punishment, rats received bilateral infusions of 0.9% phosphate-buffered saline or of the AMPA antagonist NBQX (1 µg/µl; Sigma-Aldrich, Sydney, Australia) to assess the role of LHb in the acquisition of punishment. This dose of NBQX is sufficient to prevent aversion-related signals exciting the LHb [16] and has also yielded behaviourally significant yet specific effects in previous experiments [17]. For microinjections, a 33-gauge microinjection cannula (Plastics One, Roanoke, VA, USA) was inserted into the guide cannula and connected to a 10 µl glass syringe (Hamilton Company, NV, USA) operated by an infusion pump (World Precision Instruments, FL, USA). The microinjection cannula projected a further 1 mm ventral to the tip of the guide cannula. Drugs were infused at a rate of 0.25 µl/min over 2 min, and the microinjection cannula was left in place for a further 1 min to permit diffusion of the injectate.

#### Punishment expression

On Days 6–7 all rats received bilateral infusions of either saline or NBQX (counterbalanced within-subject) to test for the effect of LHb inactivation on expression of punishment.

#### Ion channel blockers into LHb on punishment expression

On Days 9 and 11, rats (n = 10) received, in a fully counterbalanced manner, bilateral LHb infusions of Na^+^ channel blocker bupivacaine (5 µg/µl) or Ca^2+^ channel blocker mibefradil (1 µg/µl) immediately before punishment sessions. This was to assess whether blocking other means of signal transmission through the LHb would affect expression of punished and unpunished lever-pressing. The dose of bupivacaine used is sufficient to block voltage-gated Na^+^ channels [18], while the dose of mibefradil used would have effectively blocked both L- and T-type Ca^2+^ channels [19], both of which are found within the LHb [20], [21]. Rats received standard punishment sessions with no infusions on Days 8 and 10.

#### Choice test

Alternatively, instead of receiving ion channel blockers, 6 rats received a choice test on Days 9 and 11. This involved both levers being extended for 30 mins. Responses, regardless of which lever was pressed, were rewarded on a VI-60 s. No shocks were delivered. This meant that exclusively pressing the punished or unpunished lever, or a combination of both, would yield no advantage in terms of reward delivery. Rats were tested twice, once after bilateral infusions of NBQX and once after infusions of saline (within-subject, counterbalanced). Between the two choice tests, rats received a reminder punishment session, under the same conditions as the previous punished sessions, to reduce any effects the initial non-punished session might have had on performance or lever preference.

#### Locomotor Test

On Day 13, rats were placed in locomotor chambers for 40 min to habituate them to the chamber. Days 14 and 15, rats received bilateral LHb infusions of saline or NBQX (counterbalanced, within-subject) immediately before being placed into the locomotor chambers for 40 mins. Total distance travelled and velocities were measured.

#### Histology

At the end of the experiment, the rats were injected i.p. with sodium pentobarbital (100 mg/kg) and their brains were removed. Unfixed brains were quickly frozen and sectioned coronally (40 µm) through the LHb using a cryostat (Microm 560, Germany). Each section was collected and subsequently stained with cresyl violet for histological examination. The boundaries of the LHb were determined according to a reference atlas [22].

### Data analysis

The dependent measures were total punished and unpunished lever-pressing per session and latency to lever-press. Between x within-subjects ANOVAs were used to analyse lever-press training and punishment acquisition (Phase I) data, with lever (punished vs. unpunished) and day (for punishment acquisition, using linear contrasts) as the within-subjects factors, and drug group (Saline vs. NBQX) as the between-subjects factor. Within-subjects ANOVAs were used to analyse lever-presses and lever-press latencies for punishment expression (both NBQX and ion channel blockers), aversive choice and locomotor test (Phase II, III and IV). In these analyses lever (punished vs. unpunished) was one within-subjects factor and drug (saline vs. drug) was the other. All ANOVAs tested planned contrasts. Lever-press ratios were analysed using a one-sample t-test, using 0.5 (no change in lever-pressing after drug compared to after saline) as the test value. For all analyses, type I error rate (a) was controlled at 0.05.

Rats were excluded from all analyses if the cannula tip was not bilaterally located within the LHb. One rat was excluded from analysis of punishment expression due to malfunction in shock delivery isolated to its saline session, but was included in later analyses.

## Results

### Histology

**Figure 1:**
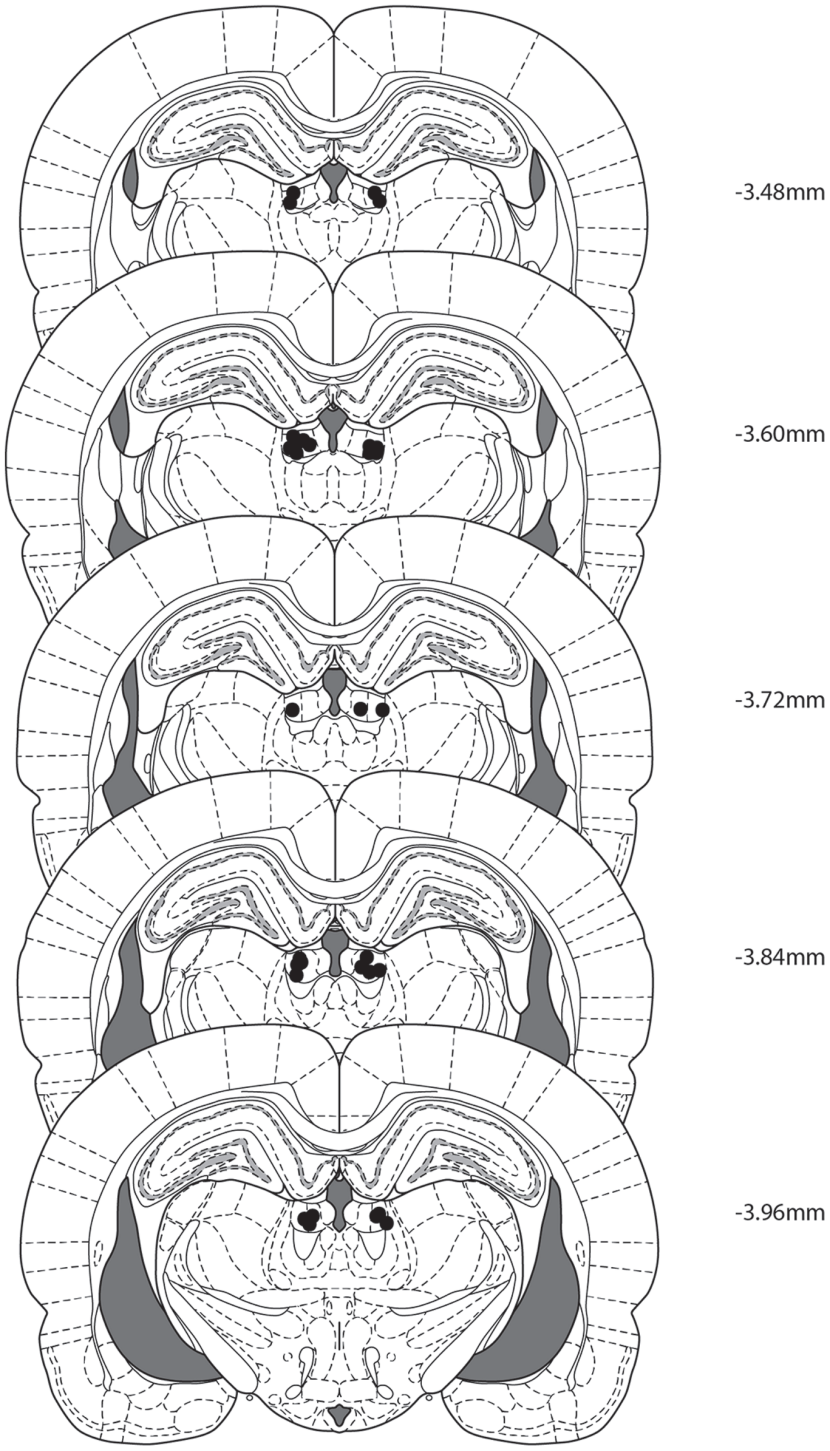
Microinfusion cannula placements within the LHb as verified by Nissl-stained sections. Black dots represent the most ventral point of the cannula tract, indicated on coronal sections adapted from Paxinos and Watson (2007).

The locations of microinjection cannulae are shown in **Figure 1**. One rat was excluded due to incorrect cannula placement. The remaining 16 rats had confirmed bilateral placements in LHb.

### Lever-press training

During the last day of lever-press training the mean and standard error of the mean (SEM) responses on the to-be-punished and to-be-unpunished levers were 657±137 and 572±92 for group saline and 635±104 and 552±55 for group NBQX. There was no significant overall difference between saline and NBQX groups in lever-pressing at the end of lever-press training (*F*_(1,14)_ <1; *p*>.05), no overall difference in responding on the to-be punished and to-be unpunished levers (*F*_(1,14)_ = 1.2; *p*>.05), and no group x lever interaction (*F*_(1,14)_ <1; *p*>.05).

### Effects of LHb inactivation on acquisition of punishment

In the five 40 min daily punishment sessions, rats had alternating periods of 5 min access to two levers. These levers were reinforced with food pellets via the same VI30s schedule used during lever-press training, and one of these levers was also punished on an FR10 schedule with delivery of footshock. Rats received infusions into the LHb immediately prior to the first two days of punishment training.

**Figure 2:**
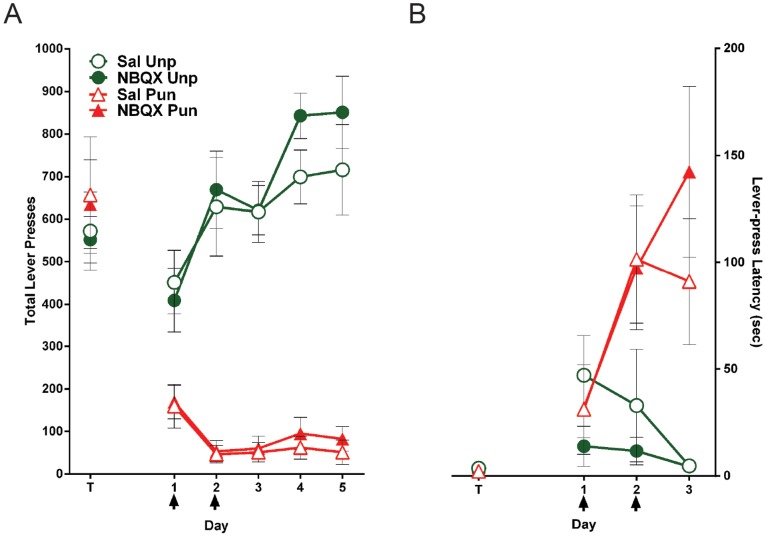
Effect of NBQX inactivation of the LHb during punishment acquisition. *(A)* Mean ± SEM lever-presses on the punished and unpunished levers prior to and during punishment acquisition. T represents the last day of lever-press training. Arrows indicate days that rats received infusions of either saline (*n* = 8) or NBQX (*n* = 8) immediately prior to the session. *(B)* Mean ± SEM latency to initially press the punished and unpunished lever (averaged across trials) during punishment acquisition.

The mean and SEM lever-presses during the punishment task are shown in **Figure 2A**. Over the course of this training, there was a significant effect of lever (punished versus unpunished), (*F*_(1,14)_ = 170.7; *p*<.05) and the difference in responding on the levers increased across days, (*F*_(1,14)_ = 36.1; *p*<.05). Across days, there was an increase in responding on the unpunished lever (*F*_(1,14)_ = 26.6; *p*<.05) and a decrease in responding on the punished lever (*F*_(1,14)_ = 15.4; *p*<.05).

Rats received LHb infusions of NBQX or saline prior to the first two days of training. During these infusion days, there was no effect of LHb inactivations using NBQX on responding to the punished (*F*_(1,14)_ <1; *p*>.05) or unpunished lever (*F*_(1,14)_ <1; *p*>.05). We also assessed latencies to emit first responses (averaged across trials) on the punished and unpunished lever during infusion days (**Figure 2B**). During these infusion days, latencies to respond on the punished lever increased (*F*_(1,14)_ = 12.0; *p*<.05) whereas latencies to respond on the unpunished lever did not significantly change (*F*_(1,14)_ <1; p>.05). There was no effect of NBQX infusions on these latencies for either the punished (*F*_(1,14)_ <1; *p*>.05) or unpunished (*F*_(1,14)_ = 1.9; *p*>.05) lever. Thus, LHb infusions of NBQX had no effect on the acquisition of responding.

### Effects of LHb inactivation on expression of punishment

At the end of training rats were tested twice, once after LHb infusion of NBQX and once after infusion of saline, for the effects of LHb inactivation on the expression of punishment. The order of these tests was counterbalanced. These tests were conducted in the same manner as the punishment acquisition sessions and so involved 5 min alternating presentations of the two levers reinforced on the same VI30s (food pellet) and FR10 (punishment) schedules as acquisition. One rat was excluded from analysis of punishment expression due to malfunction in shock delivery isolated to its saline session.

**Figure 3:**
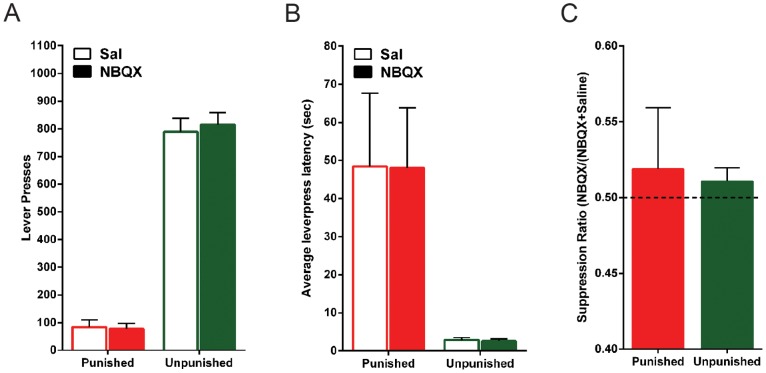
Effect of NBQX inactivation of the LHb during punishment expression. *(A)* Mean ± SEM lever-presses on the punished and unpunished levers during punishment expression. Rats received within-subject infusions of saline and NBQX (*n* = 15) immediately prior to the session, counterbalanced across days. *(B)* Mean ± SEM latency to initially press the punished and unpunished lever across trials, after infusions of saline or NBQX, during punishment expression. *(C)* Mean ± SEM suppression ratios of NBQX on lever-pressing during punishment expression.

The mean and SEM levels of performance on test are shown in **Figure 3A**. There was a significant main effect of lever, such that rats responded more on the unpunished lever than the punished lever (*F*_(1,14)_ = 292.2; *p*<.05). There was no difference in responding between NBQX and saline tests for the punished (*F*_(1,14)_ <1; *p*>.05) or unpunished (*F*_(1,14)_ <1; *p*>.05) lever.

When latencies to emit first responses (averaged across trials) were analysed, rats were significantly slower to respond on the punished lever than the unpunished lever (*F*_(1,14)_ = 14.8; *p*<.05) (**Figure 3B**). NBQX infusion into LHb had no significant effect on these latencies (all *F*_(1,14)_ <1; *p*>.05).

To further examine the effects of LHb inactivation on expression of punishment, responding on punished and unpunished levers was computed as a ratio of responding between the drug and saline tests (*ratio = A/(A+B)*) (**Figure 3C**). When this ratio equals 0.5, responding on the lever did not change between the drug and saline tests whereas values greater than 0.5 indicate an increase in responding on the drug test and values less than 0.5 indicate a decrease in responding on the drug test. NBQX did not significantly alter punished (*t*_(14)_ = .79; *p*>.05) or unpunished (*t*_(14)_ = 1.6; *p*>.05) lever-press ratios from 0.5.

### Effects of LHb ion channel blockade on expression of punishment

Following expression punishment sessions, 10 rats were subjected to an extension of the punishment expression test using counterbalanced infusions of Na^+^ channel blocker bupivacaine and Ca^2+^ channel blocker mibefradil, each immediately prior to a punishment session given 2 days apart, with an infusion-free punishment session between these two days. Responding on levers after bupivacaine and mibefradil infusions were compared to responding after saline infusions during the preceding punishment expression test (within-subject).

**Figure 4:**
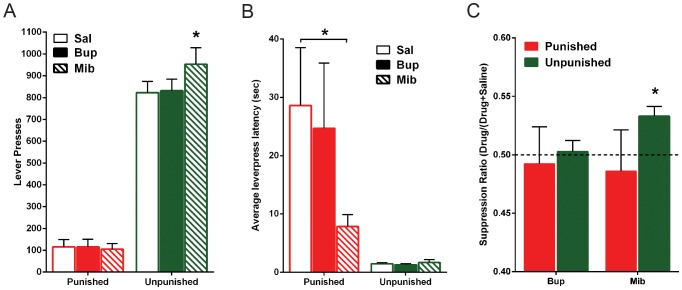
Effect of ion channel blocker inactivation of the LHb during punishment expression. *(A)* Mean ± SEM lever-presses on the punished and unpunished levers during punishment expression. Rats received within-subject infusions of bupivacaine (Bup) and mibefradil (Mib) (*n* = 10) immediately prior to the session, counterbalanced across days. *(B)* Mean ± SEM latency to initially press the punished and unpunished lever across trials, after infusions of bupivacaine or mibefradil, during punishment expression. *(C)* Mean ± SEM suppression ratios of bupivacaine and mibefradil on lever-pressing during punishment expression. * p<.05.

Mean and SEM lever-presses after channel blocker infusions are shown in **Figure 4A**. Bupivacaine had no significant effect on punished (*F*_(1,9)_ <1; *p*>.05) or unpunished (*F*_(1,9)_ <1; *p*>.05) lever-pressing when compared to saline. Mibefradil had no significant effect on punished lever-pressing (*F*_(1,9)_ <1; *p*>.05), but significantly increased responding on the unpunished lever (*F*_(1,9)_ = 14.8; *p*<.05) in comparison to saline.

Mean and SEM average latencies to emit first responses on each lever are shown in **Figure 4B**. Rats were faster to press the unpunished lever than the punished lever (all *F*_(1,9)_ >7.63; *p*<.05). Bupivacaine had no effect on these latencies (all *F*_(1,9)_ <1; *p*>.05). However, analysis of mibefradil’s effect on latencies yielded a significant drug x lever interaction (*F*_(1,9)_ = 9.6; *p*<.05). Simple effects analysis reveal that this interaction was driven by mibefradil significantly decreasing average latencies to press the punished lever (*F*_(1,9)_ = 5.2; *p*<.05) compared to saline, while no significant changes to unpunished latencies were found (*F*_(1,9)_ <1; *p*>.05). It is possible that floor effects prevented any decreases in unpunished latencies to be observed.

When suppression ratios were analysed, results concurring with total lever-presses were found (**Figure 4C**). Bupivacaine infusions had no effect on punished (*t*_(9)_ = −.24; *p*>.05) or unpunished (*t*_(9)_ = .27; *p*>.05) lever-press ratios. Mibefradil significantly increased the unpunished lever-press ratio greater than 0.5 (*t*_ (17)_ = 3.9; *p*<.05), while the punished ratio was no different from 0.5 (*t*_ (17)_ = −.40; p>.05). Thus, even though mibefradil significantly decreased latency to press the punished lever, it did not increase overall lever-pressing of that lever.

### Effects of LHb inactivation on aversive choice

Instead of receiving channel blocker infusions prior to punishment sessions, 6 rats were assessed in a choice procedure across two days that involved simultaneous presentations of both the punished and unpunished lever in 30 min sessions. There is compelling evidence that LHb manipulations may be especially effective under conditions of discrete choice [23]. In the previous tests, the punished and unpunished levers were always presented separately for 5 min epochs. Here we presented both levers simultaneously. Responding on either lever was reinforced with food pellets on a unitary VI60s schedule, but no punishment was delivered. Thus, rats were free to choose which lever to respond on and there was no benefit (i.e. in terms of possible number of rewards earned) or cost (i.e. punishment) to choosing one lever over the other. Rats received infusions prior to the two tests (NBQX and saline, counterbalanced). An infusion-free punishment session was given between these two choice task days.

**Figure 5:**
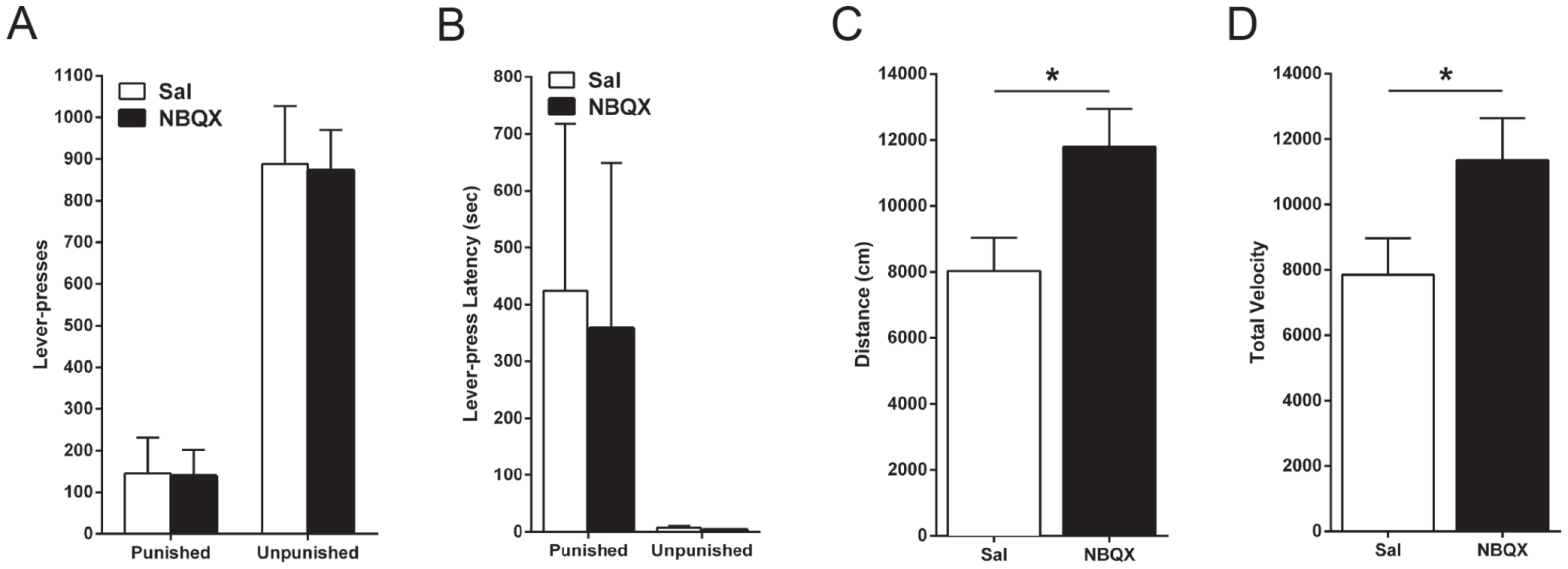
Effect of NBQX inactivation of the LHb on aversive choice and locomotion. *(A)* Mean ± SEM lever-presses on the punished and unpunished levers during the aversive choice task. Rats received within-subject infusions of saline and NBQX (*n* = 6) immediately prior to the session, counterbalanced across days. *(B)* Mean ± SEM latency to initially press the punished and unpunished lever during the aversive choice task. *(C)* Mean ± SEM distance travelled after within-subject infusions of saline and NBQX (*n* = 16), counterbalanced across days. *(D)* Mean ± SEM total velocity after within-subject infusions of saline and NBQX (*n* = 16), counterbalanced across days.

**Figure 5A** shows lever-presses on choice test and **Figure 5B** shows latencies to responses. Rats responded significantly more on the unpunished than the punished lever (*F*_(1,5)_ = 36.6; *p*<.05). There was no difference in responding between NBQX and saline tests for the unpunished (*F*_(1,5)_ <1; *p*>.05) or punished lever (*F*_(1,5)_ <1; *p*>.05). While rats were slower to respond on the punished relative to the unpunished lever, this difference did not reach statistical significance (*F*_(1,5)_ = 3.1; *p*>.05). Importantly, there was no effect of NBQX infusions on lever-press latencies (all *F*_(1,5)_ <1.2; *p*>.05). Taken together, these results show that AMPA receptor antagonism in LHb has no significant effect on aversive choice.

### Effects of LHb inactivation on locomotor activity

Lastly, rats were placed in a plain locomotor chamber for 40 mins over 2 days. They received NBQX and saline infusions immediately prior to each test (counterbalanced across days). NBQX infusions into the LHb significantly increased locomotor activity as measured by total distance travelled (*F*_(1,15)_ = 21.6; *p*<.05) (**Figure 5C**) and velocity (*F*_(1,15)_ = 18.4; *p>.05*) (**Figure 5D**).

## Discussion

This experiment studied the role of the LHb in punishment by reversibly inactivating the LHb, using NBQX, during various punishment-influenced tasks. Animals learned to reduce responding on the punished lever across the course of punishment training and the latencies with which animals responded on this lever increased. In contrast, responding on the unpunished lever increased and latencies to respond on this lever remained low. When confronted with a choice between the unpunished versus punished lever, but in the absence of any punishment, rats showed a clear preference for the unpunished lever both in terms of total lever-presses as well as latencies to respond on the two levers. LHb manipulations had subtle, and pharmacologically specific effects, on punishment. Infusions of NBQX had no significant effects on the acquisition or expression of punishment and also had no effect on instrumental choice. This was regardless of whether total responses on the levers or latencies to respond on those levers were assessed. There was also no effect of sodium channel blockade on the expression of punishment. However, calcium channel blockade did affect performances in this task. Animals treated with mibefradil were significantly faster to respond on the punished lever and also made significantly more responses on the unpunished but not punished lever. Importantly, LHb infusions of NBQX did act to increase locomotor activity – a well-documented consequence of LHb manipulations [24]–[26] – indicating that these infusions were effective in manipulating LHb.

The strongest evidence here for a role of LHb punishment is derived from the effects of the calcium channel blocker mibefradil which significantly decreased latencies to press the punished lever and also increased unpunished lever-pressing. This effect of mibefradil infusions on latencies to respond on the punished lever corresponds with previous findings that lesions of the LHb decreases latencies to step off a safe platform in an inhibitory avoidance task [14] and lesions of the fasciculus retroflexus decreases latencies to avoid an aversively conditioned goal box [12]. It also corresponds with the positive correlation between LHb activity and increased latencies to perform an unrewarded saccade [2] or escape in a learned helplessness paradigm [27]. Matsumoto and Hikosaka [28] found that electrical stimulation of the LHb after a visually guided saccade gradually increased the latency of saccades in subsequent trials. This experiment suggests LHb involvement in latency is due to calcium channel permeability and not glutamatergic excitation or sodium channel permeability, because no change in latencies were observed after NBQX or bupivacaine infusions. However, no effect on overall pressing of the punished lever was found (indeed, there were fewer total punished lever-presses after mibefradil compared to saline), suggesting initial avoidance and overall avoidance are distinct processes, of which only the former is affected by Ca^2+^ channel blockade within the LHb.

The LHb contains both L- and T-type calcium channels [20], [21] (high and low voltage-dependent calcium channels, respectively). While mibefradil is traditionally considered a T-type Ca^2+^ channel blocker, the dose of mibefradil utilised in this study would have effectively blocked both L- and T-type channels [19]. The behavioural significance of LHb Ca^2+^ channels has not yet been explored, though a recent finding by Zuo and colleagues [29] suggests that LHb Ca^2+^ channels, and not Na^+^ channels, are necessary for cocaine’s facilitation of spontaneous firing within the LHb. This cocaine-induced firing in the LHb has been linked to the aversive properties of cocaine, which involves D2 receptor feedback to the LHb from the VTA [12], [29]. Thus, the present results fit broadly with a regulatory role of LHb Ca^2+^ channels over midbrain DA and hence modulation of reward seeking behaviour by punishment.

However, the effects of mibefradil were modest, observed on only some measures, and there was no evidence here that AMPA receptor antagonism or sodium channel blockade had any effect on punishment, despite having pronounced effects on locomotor activity. The absence of such effects contrasts with the evidence for punishment-related signals found within the LHb [2] and the sufficiency of LHb stimulation in supporting punishment [8]. The reason(s) for this discrepancy is unclear. While possible that the NBQX dose utilised was only sufficient enough to increase locomotor activity but not alter punishment processing, we consider this explanation unlikely given the reasonably large dose of NBQX used throughout the experiment, with smaller concentrations of NBQX having been used to prevent pertinent basal ganglia-elicited excitation of the LHb [16]. It is also not easily attributable to differences in the nature of the aversive event (e.g., footshock versus loud noise) because the relatively weak punisher used here (0.5 mA footshock) has been shown to recruit an LHb-RMTg pathway [30]. It is likely that multiple structures and/or pathways are recruited to mediate punishment and so these other structures or pathways may have compensated for LHb inactivation. Regardless, these results suggest that the LHb may not be as critical for punishment, either acquisition or expression, as widely believed.

Of particular interest was the lack of effect of LHb manipulations on instrumental aversive choice. Stopper and Floresco [23] reported that LHb inactivation using a GABA agonist removes a rat’s preference to choose a large but risky/delayed reward over a small but certain/immediate reward. Therefore, it is possible that punishment-related signals within LHb, while sufficient but not necessary for punishment, serve a more fundamental role in decision-making. Indeed, Stopper and Floresco argued that LHb serves as a preference centre involved in decision-making. However, the findings from the choice test here suggest that this role does not extend to choice biased by previous aversive experiences. Perhaps the role of LHb in biasing decision-making is restricted to appetitive, but not aversive, modifiers of subjective value.

The most robust effect found in this study was the increase in locomotion after NBQX inactivations of the LHb. In fact, only one rat out of 16 travelled less distance after NBQX infusions compared to after saline infusions (about 8% less), but this rat was also the most active during saline test and travelled comparably far after NBQX infusions. This locomotor effect stands in agreement with many other studies showing LHb lesions or inactivations increasing locomotor activity [24]–[26], validating the manipulation within the current study. It is likely that this LHb inactivation increases locomotion by removing LHb tonic inhibition of midbrain dopamine, increasing dopamine neurotransmission in the striatum, which in turn increases locomotion [24]. The possibility that LHb inactivations interacted with aspects of the design that were not addressed by counterbalancing, such as the ongoing food deprivation (all rats were maintained at ∼90% body weight during this phase as with the previous phases), to cause the observed locomotor effects is plausible and was not directly tested in this experiment. However, it is notable that previous studies that observed LHb lesion/inactivation-induced increases in locomotion [24]–[26] did not involve food deprivation schedules. While evidently unable to affect lever-pressing within the current study, the potential confound of locomotion within previous and future studies is worth consideration.

In summary, this experiment shows that NBQX inactivations of the LHb did not affect the acquisition or expression of punishment behaviour, nor the preference to press a previously unpunished lever over a previously punished lever within an aversive choice task. Calcium channel blockade, but not sodium channel blockade, increased expression of unpunished lever-pressing while decreasing latency to press a punished lever. Finally, NBQX into the LHb significantly increased locomotion. Taken together, these results suggest the LHb is not as essential for acquiring and expressing punishment-related behaviour as previously thought. More research into the functions of the LHb is required, especially given its involvement in various disorders [7], [31], [32] and as a burgeoning treatment target [33], [34].
